# A Comprehensive Assessment of the Difficulty Level and Cultural Sensitivity of Online Cancer Prevention Resources for Older Minority Men

**Published:** 2007-12-15

**Authors:** Daniela B Friedman, Elaine K Kao

**Affiliations:** Health Promotion, Education, and Behavior, University of South Carolina; South Carolina Honors College, Interdisciplinary Studies, University of South Carolina, Columbia, South Carolina

## Abstract

**Introduction:**

Older men are at increased risk for prostate cancer. As seniors turn to the Internet for cancer information, it is important that the resources they locate about lifestyle behaviors and screening are culturally appropriate and easy to understand. This study was a comprehensive analysis of prostate cancer risk as portrayed on the Internet with assessment of content readability and cultural sensitivity.

**Methods:**

We selected Web sites about prostate cancer risk and prevention by comparing common sites across three top-rated search engines (Google, Yahoo!, and MSN). A total of 70 Web sites on prostate cancer containing a Web page on risk factors or prevention or both for racial and ethnic populations were included. We assessed readability of one page per Web site using Simple Measure of Gobbledygook (SMOG), Flesch-Kincaid (FK), and Flesch Reading Ease (FRE) measures. Cultural sensitivity of the Web page was evaluated using the Cultural Sensitivity Assessment Tool (CSAT) and questions from a cultural sensitivity checklist.

**Results:**

Mean readability of Web pages was Grade 12.90 (high school graduate level) using SMOG and Grade 11.20 according to FK. Mean FRE was 45.04 (fairly difficult to read). The mean CSAT score was 2.78 and classified as culturally sensitive. Of the 36 Web pages considered culturally sensitive (CSAT >2.50), 75% did not portray images of representative racial or ethnic individuals as intended readers or as being at high risk for prostate cancer. Older adults and seniors were identified as intended readers on 73% of Web pages.

**Conclusion:**

Online cancer resources are targeting appropriate age groups (high-risk older adults). However, the pages required fairly high-level reading skills and had limited cultural sensitivity. These factors make the pages unsuitable for diverse Internet users.

## Introduction

Prostate cancer is a leading cause of death among men in the United States, with an estimated 27,050 deaths expected in 2007 (1). Mortality from prostate cancer among black men (65.1 deaths per 100,000 black men) is over two times higher than that for whites (26.7 deaths per 100,000 white men). Older adults require accurate, reliable, age-relevant, and culturally sensitive information about prevention because they are at increased risk for chronic diseases such as cancer (1). Communicating prostate cancer prevention to older adults with the intent that they will act on the information to prevent disease is essential for healthy aging. This is a challenging task, however, because of the complexity of the information itself and the often conflicting medical reports regarding the benefits and efficacy of screening examinations. In a recent review of prostate cancer screening guidelines, researchers found that data supporting the efficacy of prostate-specific antigen (PSA) testing are not entirely compelling and that screening should not include men at average risk for prostate cancer if they are younger than 50 years of age or older than 75 years of age (2). The lack of consensus on prostate cancer screening recommendations is evident in the variable quality of online resources (3,4).

Along with such varying descriptions of screening guidelines for prostate cancer, the reading level of cancer information often is high (5,6). Therefore, it is not surprising that older men assume a passive role in their health care. Specifically, men with prostate cancer often defer treatment decisions to physicians and family members (7). These men need to receive clearer information about prostate cancer prevention so that they are better informed when making personal health and lifestyle decisions.

Despite being the leading incident cancer among men (1), awareness and coverage of prostate cancer in the mass media is limited compared with that for breast cancer, the leading incident cancer among women (5,8). Inadequate communication may reflect fewer advocacy groups for prostate cancer and reluctance of men to be vocal about an illness linked to sexuality. One study conducted with men aged 38 to 80 on their perceptions of prostate cancer screening found that older participants were especially concerned about their sex life if ever diagnosed with prostate cancer (9).

Cancer prevention messages in mainstream media rarely frame cancer content in an age-specific or culturally tailored manner that would inform diverse seniors about preventive health actions (5,8). Individuals may consider information about cancer to be irrelevant if it does not include their cultural and spiritual beliefs and attitudes about disease (10). One key recommendation to improve cultural suitability of resources is to involve stakeholders and lay people from targeted minority communities in the development and evaluation of cancer resources (11). Unfortunately, health and media organizations may not have the resources or time available to tailor or to pretest health messages for difficult terminology or cultural inappropriateness when the information must be disseminated in a timely manner.

We must consider literacy levels of intended Internet end users in the development and posting of online cancer information. More than 75 million adults have basic or below basic literacy abilities and are unable to understand materials such as prescription labels or hospital consent forms. Results of the most recent National Assessment of Adult Literacy survey showed that 34% of adults aged 50 to 64, and 59% aged 65 or older, had below basic or basic levels of literacy (12). In the context of this research on the reading level of health resources for minority men, this is especially alarming. Specifically, 67% of blacks have basic or below basic literacy skills compared with 32% of whites (13). Men also have lower literacy skills than women. Online cancer information often is written at high reading levels and is difficult for average readers or individuals with poor literacy skills to understand (6,14,15). Despite this finding, interviews with breast and prostate cancer patients showed that they prefer the Web as a source of disease information, social support, and personal stories about the cancer experience (16). Being able to use the Internet and access this information provided them with feelings of competence and control.

More than 50% of African Americans searched for health information online in 2000 (17). Close to 45% of African Americans who are online report that the Internet helps them get health care information, compared with 35% of whites (17). In one study with breast cancer patients, receipt of overall and tangible social support through the Internet was significantly higher among minority women (black and Hispanic) than among white female users (18). Furthermore, an Internet health intervention at churches for both African American men and women resulted in improved nutrition. Both nutrition and physical activity improved when the Internet intervention was combined with support within the church (19). Research on black men's use of the Internet for cancer prevention information has not been conducted.

A number of studies have been conducted on prostate cancer patients' involvement in treatment decision making (20-22), and health literacy has been examined in the context of late-stage diagnosis and disease treatment (23-25). One study showed that lower prostate cancer knowledge among patients was associated with lower literacy scores, indicating that low literacy may affect patient understanding of the treatment decision-making process (24). However, health literacy and cultural sensitivity have not been systematically explored in online information about prostate cancer prevention.

Objectives of this study were twofold: 1) to assess the reading level of prostate cancer prevention resources on the Internet that are intended for minority men and 2) to evaluate the cultural sensitivity of prostate cancer prevention information on the Internet. This is the first study to examine both the readability and cultural sensitivity of prostate cancer prevention information across a sizable number of Web sites. Other studies have examined readability of multiple cancer types (e.g., breast, colorectal, prostate) on fewer sites (6,14), or included some cancer Web sites in a larger analysis of general health resources on the Internet (13). Cultural sensitivity of cancer information on the Internet has not been comprehensively assessed. Data obtained from this research on existing Internet resources on prostate cancer will help contribute to the development, implementation, and evaluation of a culturally appropriate education program to enhance the health literacy of older black men at risk for prostate cancer.

## Methods

### Web site selection

On January 25, 2007, we selected consumer-oriented prostate cancer Web sites that are accessible through the three top Internet search engines (Google, Yahoo!, and MSN, as identified by Nielsen ratings [26]). This search strategy has been employed in previous Internet research (6,14) because most people locate health information using search engines (27). The search terms used were *prostate cancer *in combination with *risk*, *prevention*, or *screening*. A Web site was excluded if it 1) was not operational at the time of the search; 2) was a directory or provided only links to other Web pages; or 3) was not intended for consumers (e.g., research library, health care professional Web site). Although individuals express the desire for accurate and reliable health information on the Internet (including medical center Web sites and research-based resources) (28), they most often use search engines to find information that links to Web sites for commercial products. Therefore, we included commercial Web sites for analysis.

We compiled a comprehensive list of Web sites from each search engine. The top-ranking 70 Web sites from each search engine were scored, in which ranking first on a search engine was awarded 70 points, and ranking 70th on a search engine was awarded 1 point. Average scores were tallied for each of the ranked Web sites across all search engines. The 70 Web sites with the highest overall ranking across the three search engines were selected for analysis.

### Readability and cultural sensitivity testing of Web pages

Web pages identified by the search engines were opened to the Web site's home page. The first Web page within the Web site mentioning minority groups as intended readers or as high-risk groups for prostate cancer was selected for readability and cultural sensitivity analysis. The first page was identified either by clicking on links from the home page or by searching the site for prostate cancer information. The three readability measures we used were Simple Measure of Gobbledygook (SMOG), Flesch-Kincaid (FK), and Flesch Reading Ease (FRE) (29-31). SMOG is conducted on 10–30 sentences in a sample of writing and measures difficulty of content by the number of polysyllabic words. If the Web page being analyzed had 10–30 sentences, all sentences were included. If the Web page contained >30 sentences, readability was determined from the first 30 consecutive sentences on the page. FK and FRE scores were determined using tools available in Microsoft Word 2003. The score derived from the FRE formula, referred to as the FRE scale score, ranges from 100 (very easy to read) to zero (unreadable). The FK formula is a modified version of the FRE that generates a school grade-level score to indicate the education level needed to understand the material. SMOG is estimated to test for 100% comprehension; Flesch tests for 75% comprehension of the material (32).

We evaluated the cultural sensitivity of the 70 Web pages using the Cultural Sensitivity Assessment Tool (CSAT) (33). The CSAT scale ranges from 4 (strongly agree that the information is culturally sensitive) to 1 (strongly disagree that the information is culturally sensitive) on three format questions (category 1), 11 message questions (category 2), and 16 visual message questions (category 3). Scores calculated for each of the three categories are then averaged for the overall CSAT score. Print materials with overall scores of ≤2.50 are classified as culturally insensitive. The CSAT was selected because it is the only published instrument for the numeric assessment of the cultural sensitivity of cancer materials. It has not been validated in the literature, has not been previously used on Web-based cancer information, and is not intended for minority groups other than African Americans. Therefore, we also used a cultural sensitivity checklist for a more comprehensive assessment (34). Checklist questions included the following:

- Is the intended racial or ethnic group mentioned? (Directly? Indirectly?)
- Is the racial or ethnic group described as a high-risk group for cancer or as the intended readers of the cancer information?
- Does the information address the perceptions of cancer risk in the intended racial or ethnic group?
- Are complementary and alternative medicines presented as acceptable methods of cancer prevention or treatment?
- Are these cancer prevention or treatment options presented in a manner that is understandable and appropriate for the intended readers?
- Are mobilizing information (i.e., information allowing the reader to contact someone for more information) or cues to action provided?
- Is the contact person or the organization that is identified as a source of information of the same racial or ethnic group as the intended readership?
- Is the cancer message linked to credible and accessible sources?

Web pages were read thoroughly and coded independently by the researchers for a number of variables. Domains were coded as .org, .com, .gov, .edu, or other. Authorship was coded as Web site writer, freelancer, or wire service. The Web page focus was coded as risk factors, screening, or lifestyle. Readability was coded using SMOG, FK, and FRE. Cultural sensitivity was coded according to CSAT and the cultural sensitivity checklist. Also coded were the date the resource was posted or reviewed, the presence of visuals, the target minority, and the target age group. These factors have been coded in previous research (5,6,32). Readability and CSAT values were analyzed using nonparametric tests (frequencies, chi-squares; Mann-Whitney U and Kruskal-Wallis rank measures for readability data). Significance was set at *P* < .05. We also noted representative terms from the Web pages to determine the tone of prostate cancer risk messages and to provide a more complete description of the framing of prostate cancer information on the Internet.

## Results

### General description of Web sites and Web pages

Most of the 70 Web sites had domains of .com (35 [50%]) and .org (26 [37%]). Fewer Web sites had domains of .gov (3 [4%]) or .edu (2 [3%]). The average number of clicks from the home page to the Web page used for this analysis was 2.1.

We observed three main areas of focus on Web pages: risk factors, lifestyle behaviors, and screening. Most pages focused on both risk factors and lifestyle (17 [24%]), followed by risk and screening (15 [21%]), risk (13 [19%]), screening (12 [17%]), and lifestyle (8 [11%]). Three pages covered all three topics, and two pages covered both screening and lifestyle.

One-quarter of Web pages did not specify the age of intended readers. Another one-quarter mentioned middle-aged adults (30–49 years), older adults (50–64), and seniors (65 or older). The next most common age groups mentioned were both older adults and seniors (16 [23%]), followed by all ages, seniors only, middle-aged or older adults, and middle-aged adults alone. Most references to age were in the middle of the page (27 [39%]) and in introductory paragraphs (26 [37%]).

Web pages were also coded for references to minority groups (e.g., black, white, Asian, Hispanic). Both blacks and whites were mentioned most often (29 pages [41%]), followed by whites, Asians, and blacks (10 pages [14%]). Few pages (7 [10%]) discussed risk of prostate cancer among whites alone. Blacks alone, Asians alone, and whites, blacks, and Hispanics together were mentioned on one page each. Ten pages did not mention explicitly specific minorities at risk for prostate cancer, although they stated that certain races or ethnicities were at higher risk for prostate cancer.

Presence or absence of contact information was also recorded. No organizational contact information was provided on 29 (41%) Web pages. Links to other Web sites appeared on 28 (40%) pages. The remainder had multiple types of contact information including Web site links, telephone numbers, and addresses.

### Readability and cultural sensitivity of online prostate cancer resources

The mean readability score of the cancer Web pages was Grade 12.90 (95% confidence interval [CI], 12.35–13.45) using SMOG and Grade 11.20 (95% CI, 10.75–11.64) according to FK. Mean FRE was 45.04 (95% CI, 41.98–48.11) (difficult to read). Reading grade level differed by domain type, with the level being higher for .edu pages than for .gov pages. Differences were significant according to FK (Χ^2^ = 10.26, 4 *df*, *P* = .04). Table 1 presents readability scores by domain type.

Although not significant, differences in reading grade level were apparent according to Web page focus (Table 2). For instance, pages on lifestyle (diet and physical activity) were hardest to read according to SMOG, FK, and FRE measures. Pages that included both risk factor and lifestyle content were easiest to read according to SMOG, and pages on all three topics (risk factors, screening, lifestyle) were easiest to read according to FK and the FRE scale.

Samples of technical language from Web pages written at more difficult reading levels included these two examples:

Prostate biopsy prompted by abnormal findings on digital rectal exam (DRE), such as nodularity or induration of the prostate leads to a diagnosis of prostate cancer in only 15%–25% of cases. This compares with prostate cancer prevalence of less than 5% among men of similar age without abnormal DRE. Although neither accurate nor sensitive for prostate cancer detection, abnormal DRE is associated with a 5-fold increased risk of cancer present at time of screening. (SMOG for rest of Web page = 14.57; http://www.cancer.med.umich.edu/prevention/ prostate_cancer_detection.shtml.)

The research team reported that the gene seems to contribute to prostate cancer risk in a number of ethnic backgrounds, including African-American families. The study suggests that approximately 1 in every 500 men possesses an altered version of the gene. Researchers estimate that alterations in the HPC-1 gene are responsible for at least a third of familial prostate cancer, which accounts for about 1 in 10 cases of the disease. Scientists were optimistic that the HPC-1 gene may help unlock the mystery of why African-American men are exceptionally vulnerable to the disease. (SMOG for entire Web page = 14.06; http://prostateaction.org/diagnosis/lethal.html.)

Samples of easier, plain language information included these two:

Prostate cancer is more common in some racial and ethnic groups than in others, but medical experts do not know why. Prostate cancer is more common in African-American men than in white men. It is less common in Hispanic, Asian, Pacific Islander, and Native American men than in white men. (SMOG = 9.33; http://www.cdc.gov/cancer/prostate/publications/decisionguide/ index.htm#diagnosis.)

Your doctor may examine your prostate by putting a gloved, lubricated finger a few inches into your rectum to feel your prostate gland. This is called a digital rectal exam. A normal prostate feels firm. If there are hard spots on the prostate, your doctor may suspect cancer. (SMOG = 9.22; http://familydoctor.org/online/famdocen/home/common/ cancer/types/361.html.)

The mean overall CSAT score of the 70 pages studied was 2.78 (95% CI, 2.64–2.93), which is in the culturally sensitive range. Specifically, 36 (51%) Web pages were culturally sensitive (CSAT overall scores of > 2.50). A significant number of these pages (27 [75.0%]), however, did not present images of intended minorities (*t* = 3.31, 39 *df*, *P* = .002). Of the pages that were culturally sensitive and that mentioned racial or ethnic populations, all except two listed specific high-risk racial or ethnic groups. Table 3 shows the mean CSAT scores for all the Web pages by the race and ethnicity discussed on the pages. Results from the cultural sensitivity checklist found that none of the Web pages mentioned racial- or ethnic-specific perceptions of cancer risk, cultural beliefs about health, or alternative medicine.

Mean CSAT scores also differed significantly by focus (F = 2.89, 6 *df*, *P* = .02) (Table 4). The most culturally sensitive pages with the highest CSAT scores were on risk factors, screening, and lifestyle (3.26; 95% CI, 1.79–4.72). The mean CSAT score for pages on lifestyle alone was < 2.50 (2.32; 95% CI, 1.98–2.67).

Readability scores as measured by SMOG and FRE were significantly associated with "familiarity of terms" — a measure on the CSAT scale examining language difficulty of consumer health information (SMOG: Χ^2^ = 9.30, 3 *df*, *P* = .03; FRE: Χ^2^ = 8.55, 3 *df*, *P* = .04). We classified terms as familiar more often on Web pages that were easier to read.

### Message tone

Web pages were examined for cultural sensitivity and language suitability by searching for terms on tone, that is, positive or negative messages about prostate cancer and words of certainty and uncertainty regarding the link between prevention and outcomes (Table 5). Few pages used positive words or terms of certainty such as *hope*, *positive*, *proof*, or *proven*. More Web pages contained negatively charged terms such as *deadly*, *fatal*, *negative*, and *victim*. The term *evidence* as applied to prostate cancer prevention and health outcomes was used on 22 pages and mentioned 44 times, providing some assurance to readers about the associations among risk factors, prevention, and prostate cancer. Proof of such associations, however, was mentioned on only nine Web pages.

## Discussion

This study of information about prostate cancer prevention on the Internet revealed that difficult and untargeted consumer-oriented resources are being posted on the Web. While previous research showed that cancer prevention information had high reading levels (6,13,14), this is the first study to focus additionally on cultural appropriateness of Web-based resources for prostate cancer risk and prevention. As diverse groups turn to the Web for health information (35), cancer prevention resources must be culturally respectful. Although half of the Web pages analyzed were classified as culturally sensitive, one-quarter did not present images of representative racial or ethnic individuals as intended readers or as high-risk groups for prostate cancer. Many Web pages also contained negatively charged terminology, which could deter people from reading them. Culturally insensitive pages were cluttered with generic messages and images and with unfamiliar terms.

As defined by Resnicow and colleagues (36), cultural sensitivity is "the extent to which ethnic/cultural characteristics, experiences, norms, values, behavioral patterns, and beliefs of a target population's relevant historical, environmental, and social forces are incorporated in the design, delivery, and evaluation of targeted health promotion materials and programs." Cultural sensitivity consists not only of surface characteristics, such as behavioral features and appearance of the targeted population, but also cultural, social, historical, and environmental factors (that is, deep structure sensitivity) that can influence people's health behaviors and perceptions about disease prevention. Having culturally appropriate resources, which incorporate spiritual and religious beliefs as well as the importance of family and social support, has positively influenced African American men to participate in cancer education and screening programs (37,38). Use of the CSAT showed that some Web pages were indeed culturally appropriate for minority men; however, truly culturally sensitive information should include both surface and deep structure components. Results using the cultural sensitivity checklist containing items about spiritual health and cultural risk perception (34) showed that these particular aspects of health and illness are not being considered on the Web. To improve the development and usefulness of health communication materials, health resources must be created and evaluated with intended users before dissemination.

Public health educators are considering vulnerable, hard-to-reach populations in the development of prostate cancer prevention programs. For example, results from a randomized intervention for African American men found that use of an educational booklet and video led to significant increases in knowledge about prostate cancer screening compared to wait list controls (39). As we work to reduce the differences in computer and Internet access among racial groups (40), we must also ensure that information resources posted on the Web are suitable for diverse populations.

Our study presents important and original findings. First, Internet resources about prostate cancer screening were age appropriate, that is, most Web pages did mention explicitly that older men were at higher risk for prostate cancer. Previous research showed that older men are often not mentioned as intended readers or as at high risk for cancer even in publications or on Web sites specifically written for senior populations (5,6). Previous work (6) showed that compared with colorectal and breast cancer information, prostate cancer information was more often written in less technical language, though still at a high school level. This study on 70 prostate cancer Web pages showed that reading level was close to Grade 13, a difficult, college level. According to the most recent National Assessment of Adult Literacy survey (12), more than 65% of African Americans have basic or below basic literacy skills. Therefore, it is important that printed and online prostate cancer screening and prevention information be written in plain language that is understandable by all. In addition to understandable content, computers and Web pages tailored to older adults (e.g., having age-appropriate images, adequate font size, audio options for hearing-impaired) must be considered if we are encouraging seniors to seek health information on the Web. Echt and colleagues (41) stated that age-related changes in cognition (e.g., comprehension, working memory) and perceptual motor skills (e.g., task speed, motor control) can affect computer literacy development in older adults. Computer and Internet anxiety is also common among seniors. In an investigation of psychological barriers to Internet use among older adults, it was found that most seniors who had a positive perception of the usefulness, ease of use, and efficacy of the Internet used the Web more often than did those who reported negative perceptions about the Internet (42).

A surprising (though nonsignificant) finding was that resources on lifestyle behaviors were written at a more difficult level than those on screening. Post hoc analysis was conducted to see whether pages on cancer screening had less text and more images than pages on lifestyle. We found no images on lifestyle-only pages and a total of six images on pages that contained information about screening alone or with information about cancer risk. Comprehension can be affected by the extent to which the information is tailored to readers and the format in which the information is presented (i.e., text vs graphics) (43). Educational videotapes and interactive decision-making tools containing clear and relevant visuals and graphics in addition to plain language explanations have provided prostate cancer patients with greater understanding of their disease and have enabled them to participate more actively in their health decisions (44). Printed or online text alone may not meet the information needs of all consumers or patients, especially those with limited literacy or health literacy skills. The importance of plain language has been examined with respect to decision aids for prostate cancer patients. For example, plain language decision aid resources in three formats (booklet, Internet, and audio tape) were helpful to men in their decisions about localized prostate cancer treatment (45). Limited research exists, however, on the importance of literacy in understanding and engaging in prevention for prostate cancer.

Our study had several limitations. First, we consulted only 70 Web pages. Although we recognize that numerous Web sites about prostate cancer exist, we are confident that we included sites that consumers and patients find most often using three popular search engines. Second, the readability tools used have limitations. Word processing programs calculate a readability score from an estimate rather than from the actual number of syllables. Furthermore, readability formulas can produce different grade-level scores depending on the passages selected and the criterion of comprehension employed. These instruments also do not consider the influences of graphics, format, and readers' prior knowledge. Third, the CSAT tool was originally intended for printed cancer materials targeting African Americans and has not been validated in the literature. We used it, nonetheless, because it is the only available quantitative measure of the cultural sensitivity of cancer prevention resources. As discussed elsewhere (34) and as seen from our results, a limitation of the CSAT scoring system is that generic cancer articles, untailored to blacks or other minority groups, may still be rated as culturally sensitive. Finally, we did not examine quality of Web site content because it has been assessed in other research (3,4).

Guidelines for prostate cancer screening suggest that the decision to have prostate-specific antigen testing should be a shared one with physicians (46). At the same time, men report more personal control over their final decisions about screening (46). As culturally diverse individuals turn to the Web for prostate cancer information, they must be guided to clear and culturally appropriate resources to assist them with the important decision of whether or not to be screened and to encourage them to engage in healthy lifestyle behaviors that reduce the risk of developing prostate cancer.

## Figures and Tables

**Table 1:** Mean Readability Scores of 70 Web Pages Discussing Prostate Cancer Prevention, by Domain Type

Domain	SMOG^a^,^b^ Score (95% CI)	Flesch-Kincaid^b^ Score (95% CI)	Flesch Reading Ease^c^ Score (95% CI)
.com (n = 35)	12.84 (12.09–13.60)	11.10 (10.49–11.71)	45.59 (41.36–49.82)
.org (n = 26)	12.99 (11.94–14.03)	11.22 (10.47–11.98)	44.51 (39.01–50.02)
.gov (n = 3)	11.25 (6.24–16.25)	9.13 (6.58–12.68)^d^	57.63 (40.20–75.06)
.edu (n = 2)	14.88 (10.94–18.82)	14.05 (9.60–18.50)^d^	36.00 (14.40–57.60)
Other (n = 4)	12.01 (9.95–16.33)	11.93 (9.22–14.63)	38.78 (14.23–63.32)

CI indicates confidence interval.

a Simple Measure of Gobbledygook.

b Scores are presented as school grade level to indicate the education level needed to understand the material.

c Scale ranges from 0 (very difficult to read) to 100 (very easy to read).

d *P* < .05.

**Table 2:** Mean Readability Scores of 70 Web Pages Discussing Prostate Cancer Prevention, by Web Page Focus

Web Page Focus	SMOG^a^,^b^ Score (95% CI)	Flesch-Kincaid^b^ Score (95% CI)	Flesch Reading Ease^c^ Score (95% CI)
Risk factors (n = 13)	12.65 (11.22–14.08)	10.68 (9.76–11.60)	47.32 (39.86–54.79)
Screening (n = 12)	13.81 (12.09–15.52)	11.60 (10.14–13.06)	40.73 (29.05–52.42)
Lifestyle (n = 8)	14.09 (11.28–16.90)	12.13 (9.89–14.36)	39.93 (25.39–54.46)
Risk factors & screening (n = 15)	12.40 (11.33–13.46)	11.11 (10.05–12.18)	46.83 (39.94–52.35)
Risk factors & lifestyle (n = 17)	12.21 (11.65–12.77)	11.00 (10.30–11.70)	46.83 (42.28–51.38)
Screening & lifestyle (n = 2)	13.95 (6.13–21.76)	11.60 (6.52–16.68)	45.75 (6.98–98.48)
Risk factors, screening, & lifestyle (n = 3)	13.01 (2.85–23.17)	10.60 (5.81–15.39)	49.97 (34.81–65.13)

CI indicates confidence interval.

a Simple Measure of Gobbledygook.

b Scores are presented as school grade level to indicate the education level needed to understand the material.

c Scale ranges from 0 (very difficult to read) to 100 (very easy to read).

**Table 3:** Mean Cultural Sensitivity Assessment Tool (CSAT) Scores^a^ of 70 Web Pages Discussing Prostate Cancer Prevention, by Racial or Ethnic Group Mentioned on the Web Page

Racial or Ethnic Group Mentioned	CSAT Category 1 —Format Score (95% CI)	CSAT Category 2 —Written Message Score (95% CI)	CSAT Category 3 —Visual Message Score (95% CI)	Overall CSAT Score (95% CI)
General population/ white (n = 7)	3.76 (3.53–4.00)	3.26 (2.79–3.72)	0.82 (0.51–2.15)	2.63 (2.10–3.15)
Black (n = 1)	3.33	3.56	3.36	3.42
Asian (n = 1)	3.33	3.00	0.00	2.11
Black & white (n = 29)	3.64 (3.52–3.77)	3.29 (3.17–3.41)	1.73 (1.08–2.39)	2.89 (2.66–3.11)
Black, Hispanic, & white (n = 1)	3.67	3.56	0.00	2.40
Black, Asian, & white (n = 10)	3.77 (3.61–3.93)	3.27 (3.05–3.48)	1.46 (0.11–2.82)	2.84 (2.35–3.32)
>3 groups (n = 11)	3.79 (3.64–3.94)	3.47 (3.25–3.69)	1.51 (.32–2.70)	3.02 (2.61–3.44)
None (n = 10)	3.33 (3.04–3.63)	3.07 (2.74–3.40)	0.47 (−0.24 to 1.17)	2.29 (2.01–2.57)
Total (n = 70)	3.64 (3.56–3.72)	3.28 (3.20–3.37)	1.36 (0.96–1.76)	2.78 (2.64–2.93)

CI indicates confidence interval.

a The CSAT scale ranges from 4 (strongly agree that the information is culturally sensitive) to 1 (strongly disagree that the information is culturally sensitive) on three format questions (category 1), 11 message questions (category 2), and 16 visual message questions (category 3). Scores calculated for each of the three categories are averaged for the overall CSAT score. Cancer resources with overall scores of ≤2.50 are classified as culturally insensitive.

**Table 4:** Overall Cultural Sensitivity Assessment Tool (CSAT) Scores^a^ for 70 Web Pages Discussing Prostate Cancer Prevention, by Web Page Focus

**Web Page Focus**	Overall CSAT Score		
	Mean (95% CI)	Minimum	Maximum
Risk factors (n = 13)	2.69 (2.35–3.04)	1.97	3.73
Screening (n = 12)	2.54 (2.13–2.94)	1.93	3.52
Lifestyle (n = 8)	2.32 (1.98–2.67)	1.81	3.08
Risk factors & screening (n = 15)	3.18 (2.83–3.53)	1.88	3.90
Risk factors & lifestyle (n = 17)	2.79 (2.53–3.04)	2.37	3.76
Screening & lifestyle (n = 2)	2.90 (−3.58 to 9.38)	2.39	3.41
Risk factors, screening, & lifestyle (n = 3)	3.26 (1.79–4.72)	2.58	3.66
Total (n = 70)	2.78 (2.64–2.93)	1.81	3.90

CI indicates confidence interval.

a The CSAT scale ranges from 4 (strongly agree that the information is culturally sensitive) to 1 (strongly disagree that the information is culturally sensitive) on three format questions (category 1), 11 message questions (category 2), and 16 visual message questions (category 3). Scores calculated for each of the three categories are averaged for the overall CSAT score. Cancer resources with overall scores of ≤2.50 are classified as culturally insensitive.

**Table 5:** Frequency of Mention of Emotionally Charged Terms on 70 Web Pages Discussing Prostate Cancer Prevention

Term	No. of Web Pages (%)	No. of Times Mentioned
Negative	6 (9)	6
Death/Deadly	20 (29)	28
Fatal	3 (4)	3
Victim	1 (1)	1
Positive	7 (10)	17
Hope/Hopeful	4 (6)	4
Certainty	0 (0)	0
Link	11 (16)	16
Evidence	22 (31)	44
Proof/Proven	9 (13)	11
Uncertainty	2 (3)	2
Unknown	10 (14)	11
